# Impact of Intermittent Fasting and Dietary Restriction on Redox State, Energetic Metabolism, and Liver Injury in Common Bile Duct Ligation Model

**DOI:** 10.3390/antiox13070835

**Published:** 2024-07-12

**Authors:** Dmitry S. Semenovich, Ljubava D. Zorova, Polina A. Abramicheva, Nadezda V. Andrianova, Andrey V. Elchaninov, Aleksandra S. Petrukhina, Irina B. Pevzner, Vasily N. Manskikh, Dmitry B. Zorov, Egor Y. Plotnikov

**Affiliations:** 1 A.N. Belozersky Institute of Physico-Chemical Biology, Moscow State University, 119992 Moscow, Russia; 7emenovich@gmail.com (D.S.S.); ljuzor@belozersky.msu.ru (L.D.Z.); abramicheva.polina@gmail.com (P.A.A.); andrianova@belozersky.msu.ru (N.V.A.); pevzner_ib@belozersky.msu.ru (I.B.P.); manskikh@mail.ru (V.N.M.); 2 V.I. Kulakov National Medical Research Center for Obstetrics, Gynecology and Perinatology, Ministry of Healthcare of Russian Federation, 117198 Moscow, Russia; 3 Institute for Artificial Intelligence, Lomonosov Moscow State University, 119992 Moscow, Russia; 4 Avtsyn Research Institute of Human Morphology of Federal State Budgetary Scientific Institution “Petrovsky National Research Centre of Surgery”, 117418 Moscow, Russia; elchandrey@yandex.ru; 5 K.I. Skryabin Moscow State Academy of Veterinary Medicine and Biotechnology, 109472 Moscow, Russia; alepetrukhina@gmail.com

**Keywords:** liver fibrosis, bile duct ligation, dietary restriction, intermittent fasting, glucose metabolism, glycolysis, Krebs cycle, oxidative stress, redox balance, glutathione system

## Abstract

The aim of this work was to test whether we can treat cholestasis with dietary approaches applied after the onset of the disease. The effects of intermittent fasting and dietary restriction on liver damage caused by common bile duct ligation (BDL) in rats were studied, with particular attention paid to changes in the activity of enzymes of energy metabolism and antioxidant protection. Morphological changes in liver tissue and serum markers of liver damage were assessed in rats with BDL kept for one month on ad libitum diet, intermittent fasting, or 35% dietary restriction. We studied parameters of glucose metabolism (activity of glycolysis and gluconeogenesis enzymes), TCA cycle, and indicators of oxidative stress and redox status of the liver tissue. Dietary restriction resulted in an increase in gluconeogenesis activity, antioxidant capacity, and autophagy activation. When implemented after BDL, none of the dietary restriction protocols reduced the level of oxidative stress, detrimental morphological and biochemical alterations, or the fibrosis progression. Thus, under severe damage and oxidative stress developing in cholestasis, dietary restrictions are not hepatoprotective and can only be used in a pre-treatment mode.

## 1. Introduction

Cholestasis, characterized by impaired bile flow, is a significant public health problem worldwide which contributes to liver injury and dysfunction [1]. While therapeutic strategies for cholestatic liver diseases are available, their efficacy remains limited, and alternative approaches to mitigate liver damage need to be explored [2]. In recent years, growing attention has been paid to the potential benefits of metabolic interventions to enhance cellular stress resistance and promote tissue regeneration. The most studied dietary interventions are intermittent fasting (IF) and dietary restriction (DR), which include a range of dietary regimes with alternating periods of fasting or reduced caloric intake [3]. These nutritional interventions have attracted considerable interest due to their suitability for treating various diseases, including liver pathologies.

It should be noted that most studies address the effect of IF or DR in a preventive mode [4,5,6,7] when applied before a damaging stimulus. In these cases, the positive effects of the diets are shown repeatedly, including for the liver. However, the possibility of using these diets after the induction of cholestasis has not yet been investigated.

With an understanding of the molecular mechanisms underlying the protective effects of IF or DR, novel therapeutic strategies could be developed to ameliorate cholestatic liver damage and improve patient outcomes. The positive effects of IF/DR are known to be mediated by the activation of signaling pathways (AMPK, mTOR, PPARγ, etc.), usually leading to significant restructuring of cellular metabolism, activation of autophagy, and improved mitochondrial functioning [8].

Activation of these signaling pathways may contribute to increased tissue tolerance to damage. However, the effects of diet on the long-term consequences of liver damage, such as fibrosis, are largely unknown. At the same time, it is known that fibrosis progression depends on the tissue energy metabolism and that changes in the metabolic profile can both retard and accelerate a fibrosis [9,10,11,12].

Another aspect is the high metabolic and synthetic activity of the liver, where detoxification of many xenobiotics, synthesis of serum proteins, and gluconeogenesis occur [13]. These features of liver tissue make it highly susceptible to dietary impacts. However, aspects such as glycolytic enzyme activity, gluconeogenesis, the Krebs cycle activity, and antioxidant defense in the liver under various calorie restriction regimes have not been previously studied.

The aim of this study was to investigate the possible protective effect of intermittent fasting and caloric restriction of the diet on cholestatic liver injury induced by ligation of the common bile duct in rats and to analyze changes in enzymes of energy metabolism and antioxidant defenses.

## 2. Materials and Methods

### 2.1. Animals

For the study, we used 5–7-month-old male outbred Wistar rats with body weight 300–400 g (n = 28). Rats were treated according to animal protocols evaluated and approved by the animal ethics committee of the A.N. Belozersky Institute of Physico-Chemical Biology Lomonosov Moscow State University (Protocol 115-15/09/2021 from 3 September 2021) and in obedience to ARRIVE guidelines. Rats of all experimental groups had unlimited access to water but were fed according to the experimental dietary treatment protocol. Rats were kept in a temperature-controlled environment from 18 °C to 22 °C and had 12/12 h light/dark regime.

### 2.2. BDL Model and Dietary Treatment

For 3 weeks before bile duct ligation (BDL) surgery, we defined the parameters of daily food and drinking water intake in intact rats fed ad libitum. Daily food intake in intact male rats weighing 300–400 g averaged 27 ± 3 g and average water intake averaged 18 ± 5 mL per day. The rats were housed in cages with two animals. The composition of the ad libitum diet is shown in Supplementary Table S1.

For BDL modeling, a ligature was applied to the proximal and then to the distal part of the bile duct, and the duct was cut between the ligatures under anesthesia (300 mg/kg chloral hydrate, intraperitoneally). Sham-operated animals underwent laparotomy, but without isolating, ligating, or dissecting the common bile duct. After surgery, all rats were intramuscularly administered the antibiotic cefazolin (20 mg/kg) to prevent postoperative infections.

**Figure 1:**
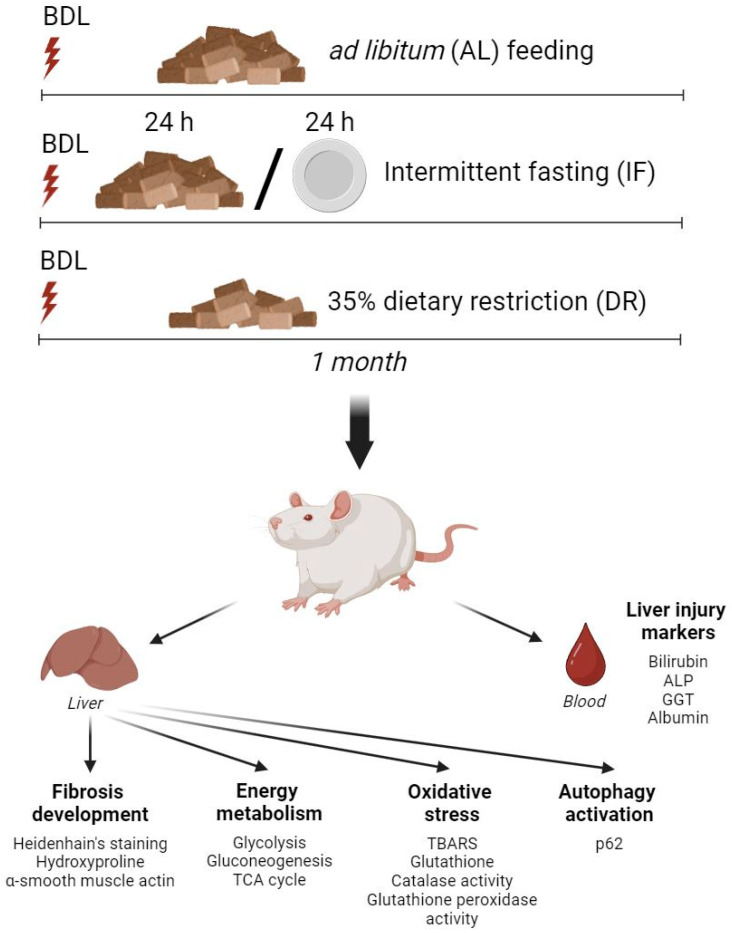
Experimental design. Experiments were carried out on rats with ligation of the common bile duct (BDL) followed by maintenance on a standard ad libitum diet (AL), intermittent fasting (IF), and 35% dietary restriction (DR). ALP—alkaline phosphatase, GGT—gamma-glutamyltransferase, TCA cycle—tricarboxylic acid cycle, TBARS—2-thiobarbiturate-reactive substances.

After BDL modeling, animals were kept on a diet restriction (IF and DR) for one month. In the case of IF, a fasting period (24 h) was alternated with 24 h of feeding ad libitum diet every day for one month. DR was performed for one month by limiting the amount of food by 35% of the daily intake. All animals had unlimited access to drinking water. Sham-operated animals kept on the standard diet ad libitum (AL) were used as a control group. The composition of a standard diet is presented in the Supplementary Materials (Supplementary Table S2). Therefore, six experimental groups were formed (Figure 1): 1—AL (n = 6); 2—IF (n = 4); 3—DR (n = 4); 4—BDL + AL (n = 5); 5—BDL + IF (n = 4); 6—BDL + DR (n = 4). The number of rats in each experimental group used for specific goals is presented in the Supplementary Table S2. Animals were sacrificed by decapitation under chloral hydrate anesthesia (300 mg/kg, intraperitoneally) to collect blood and liver tissue. After sacrificing, the livers of the rats were photographed and weighed to calculate the relative weight of the liver to body weight (in %). Liver tissue samples were frozen in liquid nitrogen and stored at −70 °C. 

### 2.3. Determination of Liver Damage Markers in Blood Serum

Blood samples were taken in tubes that contained a coagulation activator and a separating gel and incubated at room temperature for 20 min. The serum was obtained by centrifugation at 1500× *g* for 10 min, then the resulting supernatants were stored at −70 °C. The activity of alkaline phosphatase (ALP), gamma-glutamyltransferase (GGT), and the concentration of total bilirubin and albumin were determined using spectrophotometric methods (Olvex Diagnosticum, Saint Petersburg, Russia), following the manufacturer’s instructions.

### 2.4. Histological Analysis and Hydroxyproline Measurement

Tissue samples were fixed in 10% buffered formalin, washed out, and embedded in paraffin. Then, 5 µm paraffin sections were prepared, stained using Heidenhain’s Azan Trichrome method (Biovitrum, Saint Petersburg, Russia), according to the recommendations of the manufacturer, and imaged with an Axio Scope A1 microscope (Carl Zeiss, Oberkochen, Germany). Sections were examined for the presence of fibrosis in a blind fashion.

The content of hydroxyproline in liver tissue was determined via spectrophotometry using Ehrlich’s reagent [14].

### 2.5. Determination of the Activity of Energy and Glucose Metabolism Enzymes

Liver tissue samples were homogenized 1:10 (*w*/*v*) in a medium containing 50 mM Tris-HCl, pH 7.4, 150 mM KCl, 1 mM EDTA, and 0.5% (*v*/*v*) Triton X-100. Homogenates were centrifuged at 1500× *g* for 10 min, +4 °C. The supernatant was used to determine the activity of glucose-6-phosphatase, succinate dehydrogenase, cytochrome c oxidase, pyruvate, and α-ketoglutarate dehydrogenase complexes. 

The cytosolic fraction of the liver tissue was used to determine the activity of enzymes of antioxidant systems and glucose metabolism. To obtain the cytosolic fraction, liver tissue was homogenized 1:10 (*w*/*v*) in a medium containing (50 mM Tris-HCl, 150 mM KCl, and 1 mM EDTA), and then centrifuged for 30 min at 15,000× *g*, +4 °C. Samples of liver homogenates and cytosolic fraction were stored at −70 °C.

The content of total protein in the homogenate and cytosolic fraction of the liver was determined using the Lowry method [15]. Determination of glycogen content in liver tissue was carried out using the phenol-sulfuric acid method [16].

The activity of the pyruvate dehydrogenase complex (PDHC, EC 1.2.4.1), α-ketoglutarate dehydrogenase complex (αKDHC, EC 1.2.4.1) [17], and succinate dehydrogenase (SDH, EC 1.3.5.1) [18] was determined spectrophotometrically through the reduction of iodonitrotetrazolium chloride (INT) to formazan in the presence of phenazine methosulfate. Absorbance at 500 nm was measured using the INNO plate spectrophotometer (LTeck, Seongnam, Republic of Korea) in the “kinetics” mode at 37 °C in a 96-well plate. The activity of dehydrogenase complexes was calculated using the INT-formazan extinction coefficient of 12.4 mM^−1^ cm^−1^ [17]. 

Cytochrome c oxidase (COX, EC 1.9.3.1) activity was determined as the oxidation rate of reduced cytochrome c via absorbance at 550 nm with an extinction coefficient of 18.5 mM^−1^ cm^−1^ [19]. 

The activity of glucose-6-phosphatase (G-6-Pase, EC 3.1.3.9) was determined via the formation of inorganic phosphate (Pi) during the hydrolysis of glucose-6-phosphate [20]. The amount of Pi was determined using a spectrophotometric method based on the reaction with ammonium molybdate and malachite green [21]. Absorbance at 660 nm was measured on an Ekros PE-5400UV spectrophotometer (Ekrokhim, Saint Petersburg, Russia).

The activities of hexokinase (HK, EC 2.7.1.1) [22], glucose-6-phosphate dehydrogenase (G6PDH, EC 1.1.1.49) [23], glucose-6-phosphate isomerase (GPI, EC 5.3.1.9) [24], phosphofructokinase (PFK, EC 2.7.1.11) [25], and pyruvate kinase (PK, EC 2.7.1.40) [26] were determined using kinetic methods in coupled enzymatic reactions via absorbance of the formed NAD(P)H. Absorbance at 340 nm was measured in the “kinetics” mode at 37 °C.

The composition of the reaction mixtures and the protocols for determining the activity of the enzymes investigated are described in the Supplementary Table S3.

### 2.6. Determination of Parameters of Oxidative Stress and Antioxidant Systems

The basal level of 2-thiobarbiturate-reactive substances (TBARS) was determined using a spectrophotometric method based on the reaction with 2-thiobarbituric acid [27] with absorbance measured at 532 nm on an Ekros PE-5400UV spectrophotometer (“Ekroskhim”, Russia). TBARS content was calculated using a molar extinction coefficient of 156 mM^−1^ cm^−1^.

Total antioxidant activity was determined via inhibition of TBARS formation induced using Fe^2+^/ascorbate [28]. The analysis of formed TBARS was carried out as described above.

Level of reduced glutathione (GSH) in liver tissue was determined using the Ellman reagent via absorbance at 412 nm. Glutathione content was estimated using an extinction coefficient of 14.150 mM^−1^ cm^−1^ [29].

The activity of glutathione metabolic enzymes was determined in the cytosolic fraction of the liver. Glutathione-S-transferase (GST, EC 2.5.1.18) activity was determined using 1-chloro-2,4-dinitrobenzene (CDNB) as a substrate [30], with the extinction coefficient of CDNB-conjugated glutathione of 9.6 mM^−1^ cm^−1^ at 340 nm. Glutathione reductase (GR, EC 1.8.1.7) activity was determined through reduction of glutathione disulfide with NADPH production measured at 340 nm [31]. The activity of glutathione peroxidase (GPx, EC 1.11.1.9) was determined via spectrophotometric kinetic method using hydrogen peroxide as substrate [32]. The activity of glutathione synthetase (GS, EC 6.3.2.3) was determined using N-acetylcysteine as a substrate in a system of coupled reactions with pyruvate kinase and lactate dehydrogenase [33,34]. Measurements were performed at 340 nm on a Zenith 3100 plate reader at 37 °C. 

Catalase activity (CAT, EC 1.11.1.6) was determined using a spectrophotometric method based on the interaction of excess hydrogen peroxide with ammonium heptamolybdate [35]. Measurements were carried out at a wavelength of 405 nm on an Ekros PE-5400UV spectrophotometer (Ekrokhim, Russia), and 1 nmol of H_2_O_2_ consumed in 1 min at 37 °C was taken as 1 unit (U) of CAT activity.

The activity of superoxide dismutase (SOD, EC 1.15.1.1) was determined through inhibition of the autoxidation reaction of epinephrine in an alkaline medium and recording the change in optical density at 340 nm on a Zenith 3100 plate reader at 37 °C for 3 min [36,37]. The rate of 50% inhibition of the epinephrine autoxidation reaction was taken as one unit (U) of SOD activity and expressed in U/mg protein.

### 2.7. Western Blot Analysis

For western blotting, homogenized liver tissue samples 1:5 (*w*/*v*) in cold 1× PBS with 1 mM of phenylmethylsulfonyl fluoride (Amresco, Solon, OH, USA) at 4 °C. The homogenate was centrifuged at 1000× *g* for 3 min. The resulting supernatant was mixed with a 4× Laemmli sample buffer, which contained 10% 2-mercaptoethanol and then boiled at 95 °C for 5 min. To measure protein concentration, we used bicinchoninic acid assay (Sigma, Burlington, MA, USA). Samples were loaded onto a 15% Tris-glycine polyacrylamide gel to achieve 10 µg total protein per lane. After electrophoresis, the gels were transferred by rapid protein transfer using Trans-Blot Turbo (Bio-Rad, Hercules, CA, USA) onto the PVDF membrane (Amersham Pharmacia Biotech, Buckinghamshire, UK). Membranes were blocked with 5% (*w*/*v*) nonfat milk (SERVA, Heidelberg, Germany) containing 0.05% Tween-20 (Panreac, Barcelona, Spain) and incubated with the primary antibodies to SQSTM1/p62 1:1000 (#5114, Cell Signaling, Danvers, MA, USA) and α-smooth muscle actin (α-SMA) 1:1000 (ab5694, Abcam, Waltham, MA, USA). Membranes were then incubated with secondary anti-rabbit IgG antibodies conjugated with horseradish peroxidase 1:7500 (Imtek, Moscow, Russia). Detection was performed using the WesternBright Enhanced Chemiluminescence Kit (Advansta, San Jose, CA, USA) with the ChemiDoc MP Imaging System (Bio-Rad, Hercules, CA, USA). Total protein loading after electrophoresis was also monitored using TGX Stain-Free technology imaging according to the manufacturer’s instructions (Bio-Rad, Hercules, CA, USA). Specific band densities were normalized to the intensity of the TGX-staining.

### 2.8. Bioinformatic Analysis

For principal component analysis (PCA), the data were analyzed in MetaboAnalyst 5.0 software. Heatmapper (http://heatmapper.ca/, which was accessed on 9 May 2024) was used to maintain hierarchical clustering analysis. For the analysis, the mean values of the individual parameters were normalized on the mean values in the AL group and the common logarithm was performed. The Euclidean distance algorithm and the complete linkage clustering algorithm were selected to visualize the heat map.

### 2.9. Statistical Analysis

The data were analyzed in GraphPad Prism 7 (GraphPad Software Inc., San Diego, CA, USA). To calculate statistical significance, we performed parametric one-way ANOVA with Sidak’s multiple comparison test. To identify and exclude outliers, we used Grubbs’ test. Mean ± SEM are presented on the graphs; *p* < 0.05 was referred to as statistically significant; * *p* < 0.05, ** *p* < 0.01, *** *p* < 0.001.

## 3. Results

### 3.1. Effects of Dietary Restrictions on Liver Metabolism without BDL Exposure

**Figure 2:**
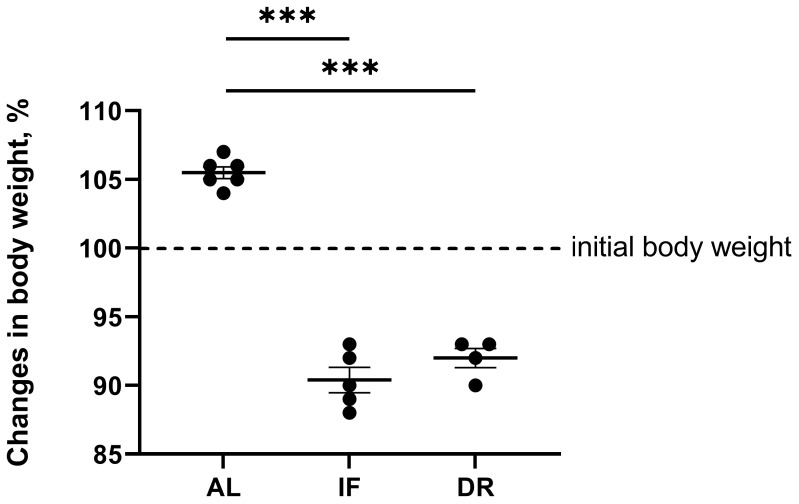
Changes in body weight of rats without BDL maintained for 1 month on ad libitum diet (AL), intermittent fasting (IF), or 35% dietary restriction (DR). The number of rats in all experimental groups was n ≥ 4. *** *p* < 0.001 (one-way ANOVA). The dots indicate the individual values of the measured parameter.

Both IF and DR for one month resulted in body weight loss (Figure 2). Body weight decreased by 10% after IF, while 35% DR resulted in a weight loss of 7% compared to the weight of the rats at the beginning of the experiment. At the same time, the rats that received an AL diet gained 5% of their initial body weight.

**Figure 3:**
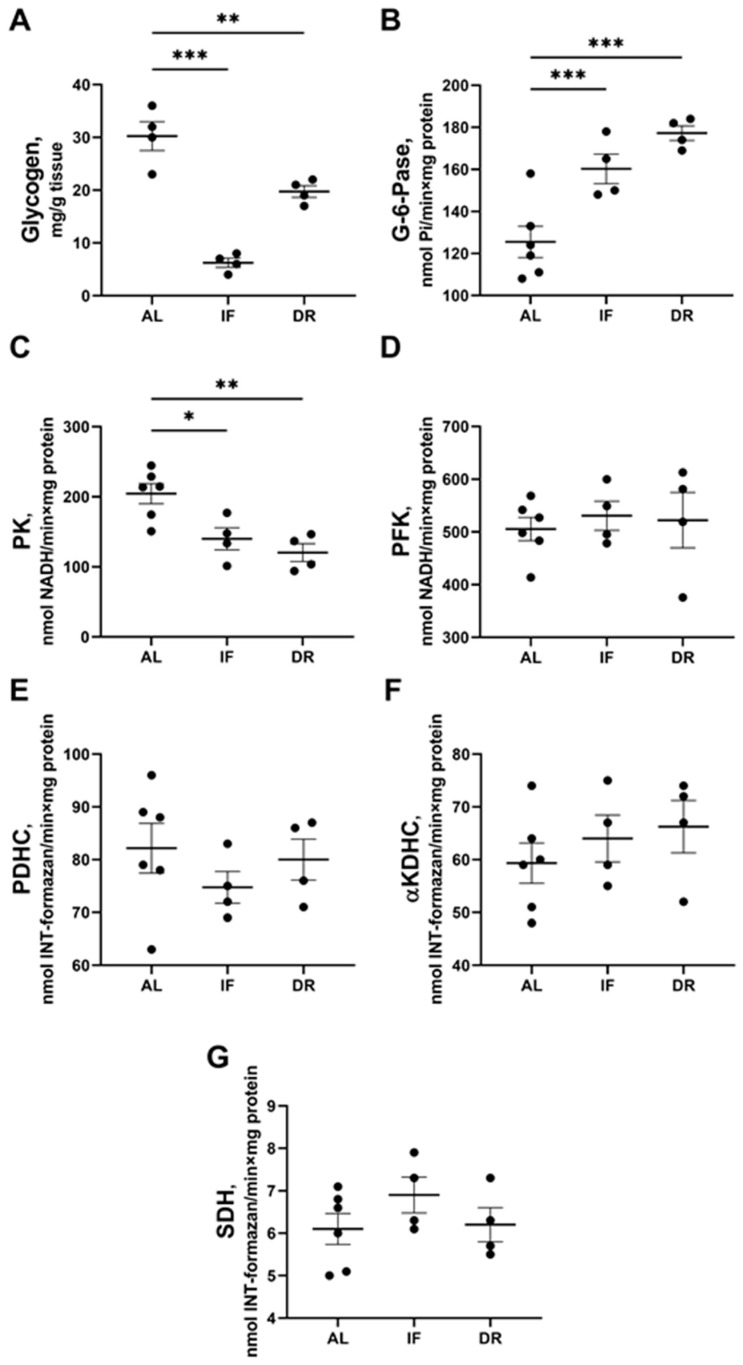
Glycogen levels and activity of key enzymes of gluconeogenesis, glycolysis, TCA cycle, and pyruvate dehydrogenase complex (PDHC) in the liver after 1 month on ad libitum diet (AL), intermittent fasting (IF), and 35% dietary restriction (DR): (**A**) glycogen levels; (**B**) glucose-6-phosphatase (G-6-Pase); (**C**) pyruvate kinase (PK); (**D**) phosphofructokinase (PFK); (**E**) pyruvate dehydrogenase complex (PDHC); (**F**) alpha-ketoglutarate dehydrogenase complex (αKDHC); (**G**) succinate dehydrogenase (SDH). The number of rats in all experimental groups was n ≥ 4. * *p* < 0.05, ** *p* < 0.01, *** *p* < 0.001 (one-way ANOVA). Each dot in a graph represents individual value of the measured parameter.

The glycogen content in the liver of the rats in the IF and 35% DR groups was reduced by 80% and 35%, respectively, which may indicate activation of glycogenolysis (Figure 3A). Analysis of the activities of glucose metabolism enzymes in liver tissue revealed activation of gluconeogenesis after IF and DR, as indicated by an increase in glucose-6-phosphatase activity (Figure 3B), while glycolysis was affected by a decrease in the activity of pyruvate kinase (Figure 3C). Glucose-6-phosphatase activity was 24% higher in the IF group and 32% higher in the DR group, compared to animals in the AL group. Liver pyruvate kinase activity was 25% and 28% lower in the IF and DR groups, respectively, while upstream glycolysis enzymatic activity s of (hexokinase, glucose-6-phosphate isomerase) did not change (Supplementary Figure S1). However, dietary restrictions did not affect the activity of mitochondrial multienzyme complexes (PDHC, αKDHC, and SDH) (Figure 3E–G).

**Figure 4:**
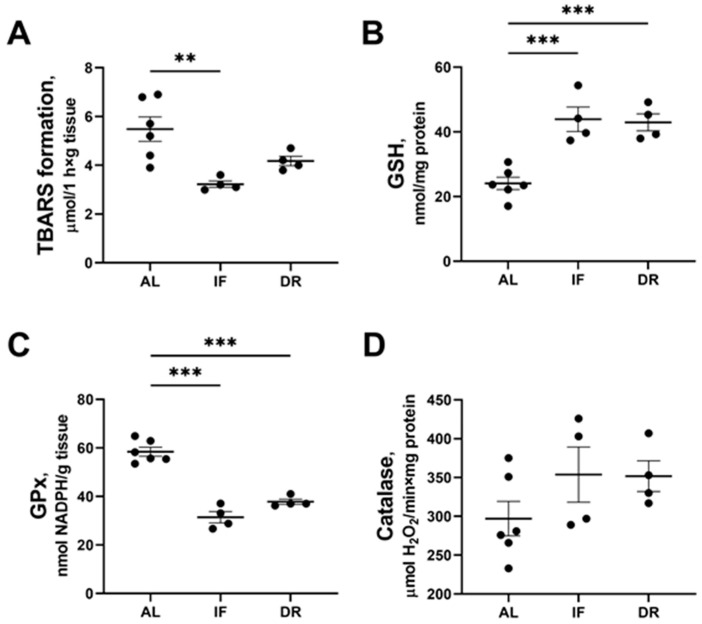
Assessment of antioxidant capacity in liver tissue. TBARS formation during Fe^2+^/ascorbate-induced oxidative stress (**A**), changes in the liver content of reduced glutathione (GSH) (**B**), an activity of glutathione peroxidase (GPx) (**C**), and catalase (**D**) in the liver of rats after 1 month on standard diet (AL), intermittent fasting (IF), or 35% dietary restriction (DR). The number of rats in all experimental groups was n ≥ 4. ** *p* < 0.01, *** *p* < 0.001 (one-way ANOVA). Each dot in a diagram represents an individual value of the measured parameter.

DR and IF were accompanied by changes in the functioning of antioxidant systems in the liver tissue (Figure 4). When assessing the total antioxidant capacity of the liver a 1.5-fold decrease in Fe^2+^/ascorbate-induced TBARS production was revealed in the IF group compared to the AL group (Figure 4A). A similar trend in TBARS production was found in the DR group. The decrease in the rate of TBARS production could be explained by a 2-fold increase in the content of reduced glutathione detected in the liver tissue of rats after IF and DR (Figure 4B). The increase in the reduced glutathione levels was accompanied by a decrease in GPx activity of more than 1.5 times in the liver of IF- and DR-exposed rats (Figure 4C). On the other hand, IF and DR did not lead to a change in catalase activity (Figure 4D).

**Figure 5:**
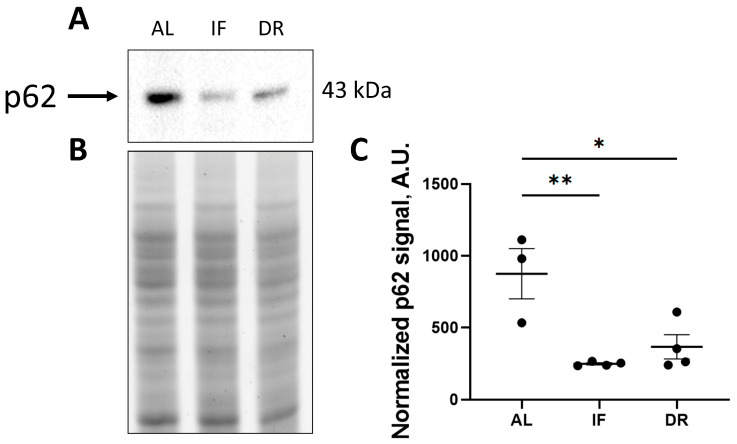
Assessment of changes in p62 protein levels in the liver of rats. Western blotting (**A**), loading control with TGX Stain-free imaging (**B**), and densitometry of the specific bands (**C**). The number of rats in all experimental groups was n ≥ 4. * *p* < 0.05, ** *p* < 0.01 (one-way ANOVA. Each dot in the diagram (**C**) represents an individual value of the measured parameter.

Evaluation of autophagy flux revealed a significant drop in p62 protein levels after both DR and IF (Figure 5). Such a 2.5-fold decrease in this marker may indicate the induction of autophagy in the liver tissue.

### 3.2. Effects of BDL and Consequent Various Diets on Liver Damage

**Figure 6:**
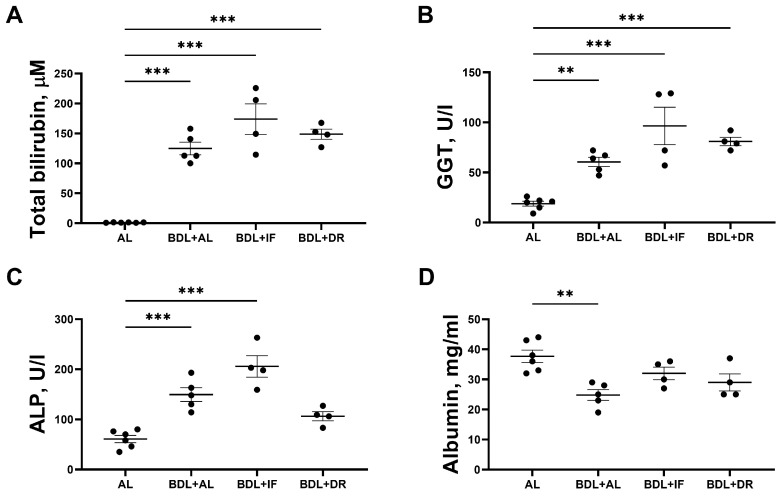
Biochemical markers of liver damage in the rat serum. (**A**) Serum total bilirubin concentration. (**B**) Serum GGT activity. (**C**) ALP activity in serum. (**D**) Serum albumin concentration. The number of rats in all experimental groups was n ≥ 4. ** *p* < 0.01, *** *p* < 0.001 (one-way ANOVA). Each dot in a diagram represents an individual value of the measured parameter.

To assess liver damage, conventional serum biomarkers were examined: the concentration of total bilirubin, albumin, GGT, and alkaline phosphatase activity (Figure 6). BDL led to the onset of obstructive cholestasis in rats in all experimental groups, which was confirmed by the development of hyperbilirubinemia (Figure 6A) and increased activity of liver enzymes in the blood serum (Figure 6B,C). All BDL animals fed on the three diets tested showed a significant increase in GGT activity (Figure 6B). ALP activity also increased in BDL rats, with the exception of the group of BDL animals kept on the DR, in which ALP activity was lower than in other BDL groups, but did not reach the values in the control group (Figure 6C). It should be noted that BDL animals receiving the restricted diet did not exhibit impaired liver protein synthesis function, as evidenced by albumin levels, while BDL animals receiving all diets exhibited hypoalbuminemia (Figure 6D). These results showed that IF and DR did not mitigate liver damage and dysfunction.

**Figure 7:**
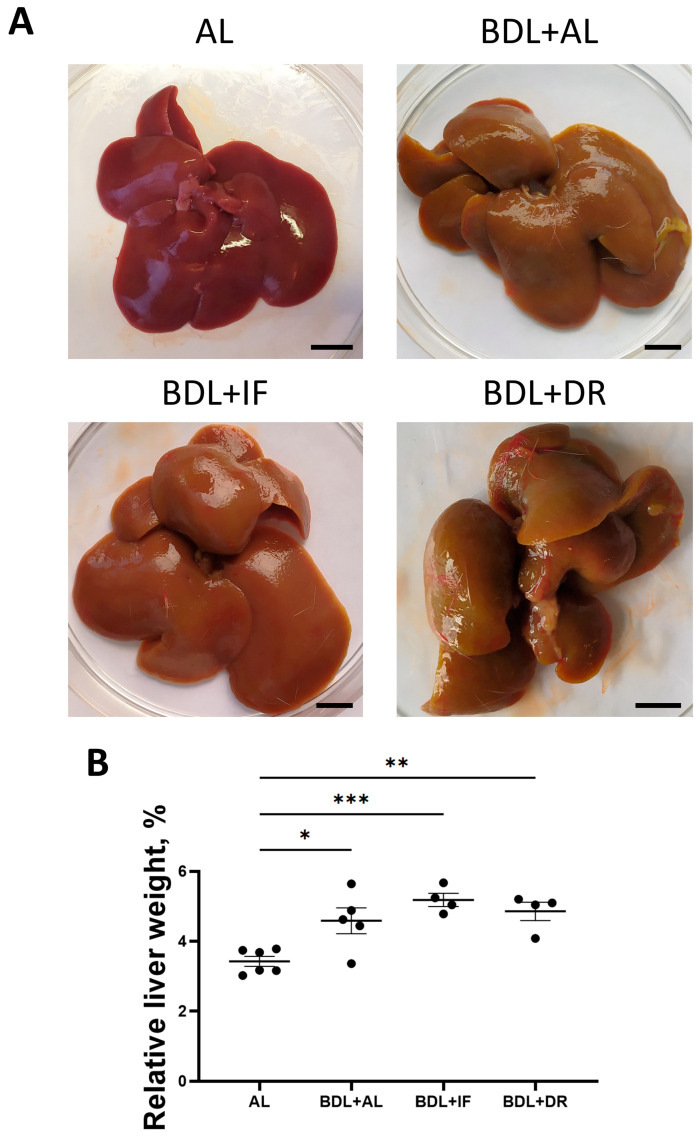
Macroscopic changes in the liver of rats with BDL and different diets. (**A**) Representative macroscopic images of the liver. Bar, 1 cm. (**B**) Relative weight of rat liver. The number of rats in all experimental groups was n ≥ 4. * *p* < 0.05, ** *p* < 0.01, *** *p* < 0.001 (one-way ANOVA). Each dot in the diagram (**B**) represents an individual value of the measured parameter.

At the endpoint of the experiment, we performed a morphological study of the liver to assess the development of fibrosis after BDL followed by various diets. The liver of sham-operated rats was brownish brown in color, without compactions, and had a smooth and shiny surface with sharp edges (Figure 7A). The appearance of the liver was significantly changed after BDL. The liver of BDL rats was yellow-brown, significantly enlarged, hardened, and had an uneven surface with small tubercles and blunt edges. The common bile duct was dilated at the ligation site to 8–10 mm and filled with yellow-green bile. To quantify hepatomegaly, relative liver weight was calculated (Figure 7B). Compared with sham-operated animals, in animals with BDL, the relative liver weight of rats was 34%, 51%, and 42% higher on the AL, IF, and DR diets, respectively.

**Figure 8:**
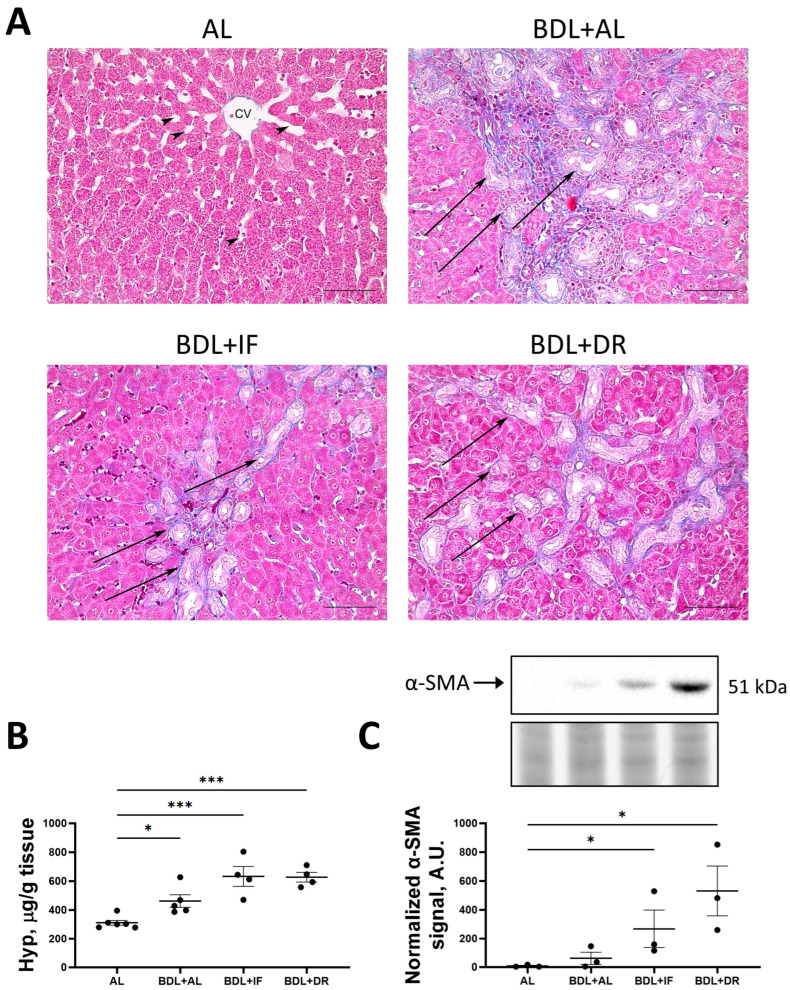
Liver fibrosis after BDL. (**A**) Staining of connective tissue with Heidenhain-azan protocol. Arrows show interlobular bile ducts. Bar, 100 µm. (**B**) Hydroxyproline (Hyp) content in liver tissue. (**C**) Change in the levels of smooth muscle alpha-actin (α-SMA), determined by western blotting and normalized to densitometry signal of total protein TGX stain-free imaging. The number of rats in all experimental groups was n ≥ 4. * *p* < 0.05, *** *p* < 0.001 (one-way ANOVA). Each dot in the diagrams (**B**) and (**C**) represents an individual value of the measured parameter.

The development of liver fibrosis after BDL was assessed on histological sections with Heidenhain-azan staining, as well as by the levels of hydroxyproline and α-SMA (Figure 8A–C). In sham-operated animals, collagen fibers were predominantly visible around the portal triads. The liver parenchyma has a characteristic structure consisting of classic hexagonal hepatic lobes. Hepatocytes form cords, between which there are sinusoidal capillaries (indicated by arrowheads), converging to the center of the hepatic lobule. In the center of the lobules are the central veins. Portal triads consisting of interlobular vein, artery, and interlobular bile duct are located at the periphery of the lobules. The boundaries between the lobules are practically indistinguishable due to the small amount of connective tissue. All BDL-exposed groups showed a slight increase in blue-stained collagen fibers and morphological changes considered as fibrosis (Figure 8A). In the liver of rats with BDL on the AL diet, the structure of the liver parenchyma was greatly altered. There was a strong proliferation of interlobular connective tissue. Numerous interlobular bile ducts (marked by arrows) were found in connective layers. Rats with BDL on IF had changes in the liver similar to those in the AL group. However, it can be noted that the proliferation of interlobular bile ducts (marked by arrows) was less pronounced. In rats with BDL on a DR diet, the liver parenchyma was significantly altered. There was a significant proliferation of interlobular bile ducts (marked by arrows), which penetrate deeply into the parenchyma of the hepatic lobules.

In BDL rats fed both AL and food-restricted diets, hydroxyproline levels were significantly higher than in sham-operated animals (Figure 8B). Moreover, IF and DR after BDL resulted in significantly higher α-SMA protein levels than AL (Figure 8C). 

### 3.3. Changes in Hepatic Energy Metabolism after BDL and Different Diets

**Figure 9:**
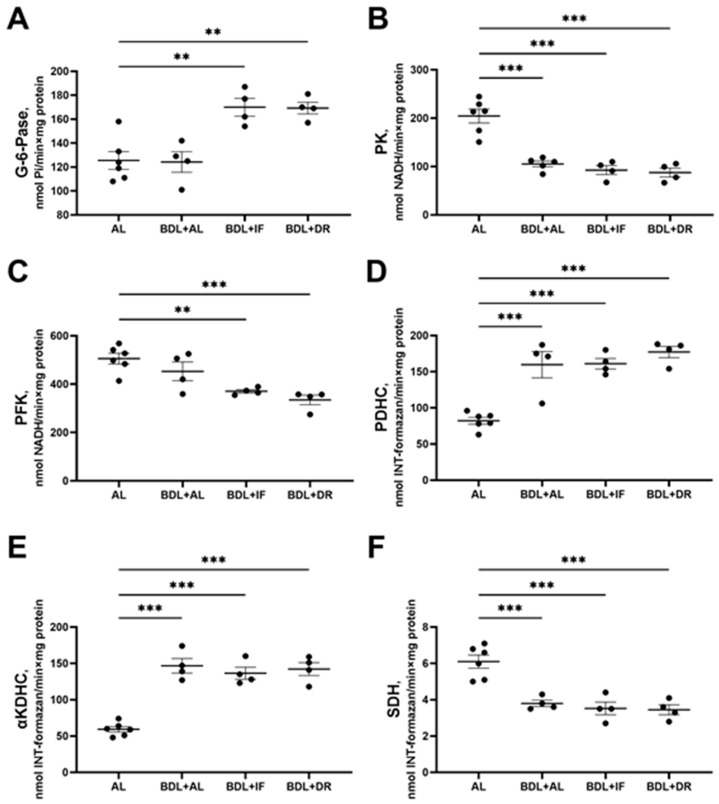
Activity of key enzymes of energy metabolism in the liver after BDL: (**A**) glucose-6-phosphatase (G-6-Pase); (**B**) pyruvate kinase (PK); (**C**) phosphofructokinase (PFK); (**D**) pyruvate dehydrogenase complex (PDHC); (**E**) alpha-ketoglutarate dehydrogenase complex (αKDHC); (**F**) succinate dehydrogenase (SDH). The number of rats in all experimental groups was n ≥ 4. ** *p* < 0.01, *** *p* < 0.001 (one-way ANOVA). Each dot in a diagram represents an individual value of the measured parameter.

IF and DR, when applied after BDL, promoted the activation of gluconeogenesis in the liver, as evidenced by a 1.4-fold increase in G-6-Pase activity in both groups compared to healthy animals on AL (Figure 9A). The activity of enzymes of glycolysis (PK and PFK) in the liver of BDL rats decreased in all experimental groups (Figure 9B,C) without effects of the diets. PK activity was 2-fold lower in animals with BDL fed both AL and IF or DR (Figure 9B). PFK activity decreased to a greater extent in animals with IF or DR after BDL (Figure 9C), while BDL alone did not result in PKF activity drop. BDL in all groups resulted in a 1.7-fold increase in PDHC activity and a 3-fold increase in αKDHC activity compared to the control group (Figure 9D,E), while SDH activity decreased 1.5 times (Figure 9F). IF and DR did not affect BDL-induced alteration in the activity of these enzymes. 

### 3.4. Oxidative Stress and Activity of Antioxidant Enzymes in the Liver

**Figure 10:**
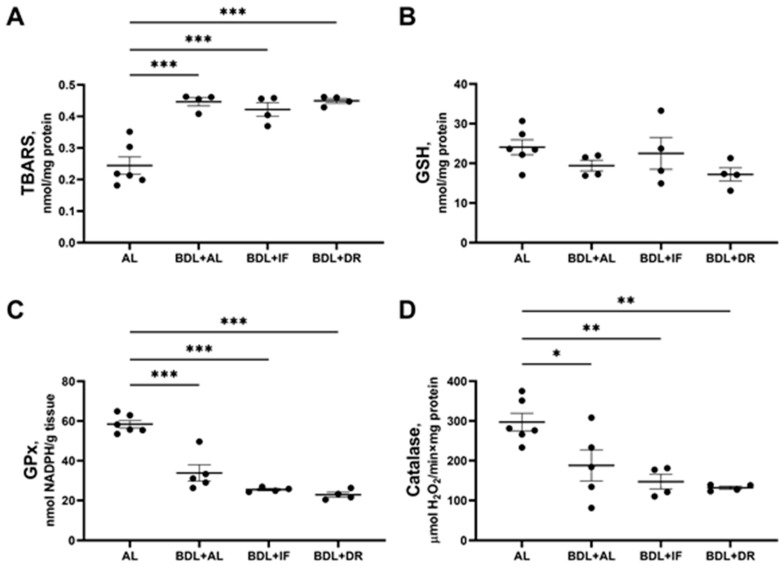
Oxidative stress and activity of antioxidant enzymes after BDL: changes in the liver content of TBARS (**A**) and reduced glutathione (GSH) (**B**), an activity of glutathione peroxidase (GPx) (**C**), and catalase (**D**). n ≥ 4 in all experimental groups. * *p* < 0.05, ** *p* < 0.01, *** *p* < 0.001 (one-way ANOVA). Each dot in a diagram represents an individual value of the measured parameter.

Animals with BDL demonstrated signs of oxidative stress development in the liver tissue (Figure 10A–D). We found an approximate 2-fold increase in TBARS levels in all groups after BDL whether dietary-restricted or fed ad libitum (Figure 10A). However, the content of reduced glutathione in the liver did not change in all three experimental groups with BDL (Figure 10B), while in animals without BDL, IF, and DR increased the reduced glutathione levels (Figure 4B above). Along with increasing TBARS levels, all animals in the groups with BDL showed significantly decreased catalase activity and glutathione peroxidase compared to the control group (Figure 10C,D). IF and DR after BDL did not lead to an improvement in antioxidant status or to a decrease in the content of lipid peroxidation products in the liver. 

### 3.5. Bioinformatic Analysis of the Metabolic Profile of the Liver

**Figure 11:**
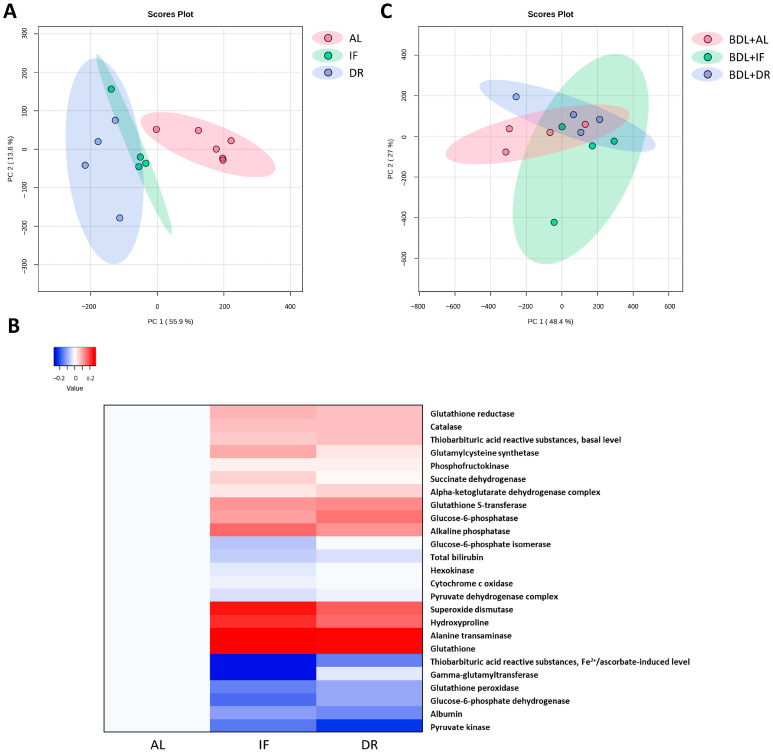
The effect of *ad libitum* diet (AL), intermittent fasting (IF), and dietary restriction (DR) on the whole set of metabolic parameters, liver function, and damage markers. (**A**) PCA of changes in blood and liver tissue parameters after different diets in healthy rats. (**B**) PCA of changes in blood and liver tissue parameters after different diets in rats with BDL. (**C**) Heat map of changes in biochemical parameters of rats after AL, IF, and DR without BDL.

Multivariate analysis of the obtained results using principal component analysis (PCA) revealed that the metabolic profile of rats without BDL kept on IF or DR diets significantly differed from control rats (Figure 11 A). At the same time, biochemical parameters in the liver of rats on IF and DR diets were mostly altered in the same way (Figure 11B). After BDL, the changes in metabolic parameters were similar in rats on all diets (Figure 11B and Figure S2).

## 4. Discussion

Dietary restriction has repeatedly proven its effectiveness as a therapeutic tool for various pathologies, as well as for life extension [38,39]. Usually, either very long periods of food restriction, as in the study of age-related changes, or temporary effects of diet on the body before the onset of pathology are used. However, for clinical practice, however, it is more valuable to use dietary interventions as therapy against the background of an already-developed disease. Therefore, in this work, we used food restriction and intermittent fasting after induction of the BDL model. 

Food restriction has been previously considered as an approach to hepatoprotection [40,41,42,43,44], but this was not done on a cholestasis model, nor was DR applied before the development of the pathology. Since the beneficial effect of dietary restriction may be based on different mechanisms that are effective in various pathologies, it was interesting to examine how DR and IF work in cholestasis. When the liver is damaged by BDL, the most important mechanism for the development of pathology is oxidative stress, since in cholestasis, the bile acids accumulated in hepatocytes modulate mitochondrial respiration and electron transport and stimulate the formation of reactive oxygen species (ROS) in liver mitochondria [45]. On the other hand, the liver is an organ in which oxidative reactions of various xenobiotics and metabolic products actively take place, which is associated with an intensive formation of free radical oxidation products in the liver cells [46]. It has been shown that the accumulation of lipid peroxidation products occurs as early as 2 days after BDL [47]. In our study, both caloric restriction regimes, IF and DR, contributed to the inhibition of TBARS formation in the liver of control rats during the induction of lipid peroxidation activated by Fe^2+^/ascorbate. This can be associated with a 2-fold increase in the level of reduced glutathione in the liver in IF and DR. Thus, outside of pathology, these diets had an antioxidant effect. However, the basal level of TBAR in the liver of BDL rats remained higher in both IF and DR and in the ad libitum diet than in the control animals. Thus, the positive changes in redox status and antioxidant capacity observed in control rats under the influence of diet are not observed in BDL rats. Likely, the increase in antioxidant status in the liver cannot be accomplished because the deleterious effects of BDL on the liver are too strong expressed in uncompensated disbalance between a high level of pre-existing oxidative stress and a decreased activity of antioxidant enzymes that metabolize H_2_O_2_ (glutathione peroxidases and catalases).

In many studies, activation of the autophagy system is considered to be the most important molecular mechanism of IF and DR. Under normal conditions, autophagy in the liver is involved in a quality control system that regulates protein turnover and organelle renewal. Cellular components to be degraded are often labeled with ubiquitin to facilitate their recognition by autophagy receptors. Under caloric restriction, AMPK phosphorylates the ULK1 complex, which initiates autophagophore formation. p62 is a ubiquitin-binding protein that binds ubiquitin residues to adapter proteins during autophagophore formation, promoting cargo degradation in autophagolysosomes [48]. In our study, we found a decrease in the expression of the p62 protein in the liver of control rats receiving IF and DR for 1 month. This indicates an activation of autophagic flux and could potentially contribute to the protection of liver tissue in animals with BDL kept on these diets. On the other hand, autophagy plays an important role in cholestatic liver injury and is activated in BDL, as evidenced by increased expression of autophagic proteins and autophagosome formation [49]. Increased levels of retained bile acids in the liver in BDL suppress autophagy processing in hepatocytes [50]. Suppression of autophagy may therefore further exacerbate damage in BDL. Conversely, induction of autophagy via rapamycin reduces oxidative stress and liver damage in BDL. In primary biliary cholangitis, autophagy is activated and the expression of p62 and LC3 is increased in biliary epithelial cells and in periportal hepatocytes surrounding the damaged bile ducts [51]. In mouse models, autophagy is activated by ligation of the bile ducts [49,52]. Mice deficient in Atg5 or Atg7 exhibit increased accumulation of p62, which correlates with more severe intrahepatic cholestasis, suggesting that defective autophagy exacerbates cholestatic liver injury [53]. The dual role of autophagy in cholestasis does not allow a clear conclusion on the importance of its activation for liver protection, which we observed varying the diets. However, since no hepatoprotection was observed in post-BDL treatment with the diets, we can assume that in this case there is no positive effect on autophagy.

The liver plays a central role in regulating the production and catabolism of energy substrates, so it was of particular interest to analyze the effects of IF and DR on various parameters of glycolysis and oxidative metabolism. In our study, we found changes in glucose metabolism with IF and DR. Specifically, in animals with BDL kept in conditions o IF or DR, activation of gluconeogenesis was observed due to increased activity of G-6-Pase, while the activity of upstream glycolysis enzymes (GK, G-6-P isomerases, aldolases) either did not change or decreased (PK and to a lesser extent PFK). The changes in gluconeogenesis could be related to the previously demonstrated effects of a restrictive diet on lipid metabolism in the liver by decreasing lipogenesis and enhancing lipolysis and ketogenesis [54], as well as the changes in sphingosine-1-phosphate signaling, carnitine biosynthesis, and shuttle pathways [55]. Interestingly, short-term DR-enhanced β-fatty acid oxidation and suppressed triglyceride synthesis, leading to improvement in non-alcoholic fatty liver disease or hepatic insulin resistance [56]. Beta-oxidation of fatty acids is not capable of producing gluconeogenic substrates, but under conditions of energy deficiency, beta-oxidation is one of the sources of energy production and acetyl-CoA, which is used for ketogenesis during prolonged fasting [57].

Apart from the molecular mechanisms mentioned above, we attribute the loss of the hepatoprotective effect of DR and IF to the fact that mild malnutrition with cholestasis could develop, which could be exacerbated by additional dietary restriction. We point out that dietary restriction is effective in selected regimens when there are no external changes in intake or nutritional deficiencies, whereas under conditions of malabsorption typical of gastrointestinal tract pathologies (such as cholestasis), additional dietary restriction could be harmful and therefore cannot be used as a post-treatment in such models. 

Further comprehensive investigation of the different diets and their molecular mechanisms is required to develop effective dietary protocols for clinical practice. In particular, it has been shown for some types of diets that their cyclic rather than constant application may be more beneficial [58]. In addition, organ metabolism is strongly influenced by the composition, concentration, and duration of the diet [59], which should be taken into account when evaluating the efficacy of dietary regimens in different disease conditions.

## 5. Conclusions

**Figure 12:**
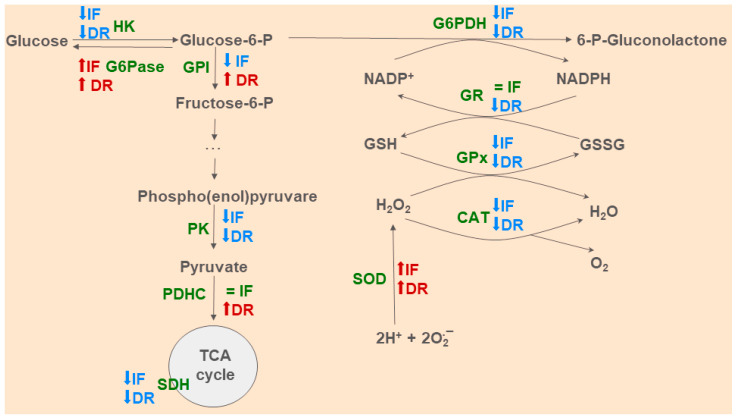
Major changes in glucose metabolic pathways and redox state of the liver induced by intermittent fasting (IF) and 35% dietary restriction (DR) during common bile duct ligation (BDL) in rats.

We found several pronounced changes in the metabolic profile and redox state in the liver caused by IF and 35% DR. The effects of both restriction diets were similar and can be interpreted as hepatoprotective. However, when used after the onset of cholestasis, these diets failed to produce beneficial effects on the liver. We attribute this to the malnutrition and severe oxidative stress developed in the BDL model making it unachievable protective effects of diets. We suppose that in this liver pathology, a better effect could be achieved by pre-exposure to diets, while when cholestasis develops, the therapeutic effect is lost. We summarize in Figure 12 the main metabolic and redox-modulating effects of IF and 35% DR in the liver after BDL.

## Data Availability

Data generated and analyzed during the current study are available from the corresponding author on reasonable request.

## Supplementary Materials

The following supporting information can be downloaded at https://www.mdpi.com/article/10.3390/antiox13070835/s1, Table S1: Composition of feed for rats for a standard diet ad libitum; Table S2: The number of rats in each experimental group; Table S3: Detailed protocols and composition of the reaction mixtures for determination of enzyme activity in rat liver tissue homogenates; Figure S1: Activity of initial glycolytic enzymes in the cytoplasmic fraction of the liver of rats kept on an ad libitum diet, intermittent fasting and dietary restriction; Figure S2: Heat map of changes in biochemical parameters in rats with common bile duct ligation on an ad libitum diet, intermittent fasting and dietary restriction.
