# Supplementary material for: Impact of Intermittent Fasting and Dietary Restriction on Redox State, Energetic Metabolism, and Liver Injury in Common Bile Duct Ligation Model
Source: Antioxidants (Basel). 2024 Jul 12;13(7):835. doi: 10.3390/antiox13070835 (PMC11273810; doi:10.3390/antiox13070835)
Supplement: Supplementary file 1 [file antioxidants-13-00835-s001.zip › antioxidants-3083149-supplementary.pdf]

Impact of Intermittent Fasting and Dietary Restriction on Redox State, Energetic Metabolism, and Liver Injury in Common Bile Duct Ligation Model

Dmitry S. Semenovich, Ljubava D. Zorova, Polina A. Abramicheva, Nadezda V. Andrianova, Andrey V. Elchaninov, Aleksandra S. Petrukhina, Irina B. Pevzner, Vasily N. Manskikh, Dmitry B. Zorov and Egor Y. Plotnikov

Supplementary File

Supplementary Table S1. Composition of feed for rats for a standard diet *ad libitum*. Manufacturer: Laboratormkorm LLC (Moscow, Russia).

Raw Protein	not less than 19%
Raw Fat	no more 5%
Raw fiber	no more 5%
Raw ash	no more 7%
Humidity	no more 10%
Vitamin A	not less than 5000 IU/kg
Vitamin D	not less than 500 IU/kg
Vitamin E	not less than 30 IU/kg
Calcium	0,9-1,2%
Phosphorus	0,6-0,9%
Sodium	not less than 0.2%
**Energy value**	**not less than 300 kcal/100 g**

Notes:
Standard diet ingredients - wheat, wheat feed flour, defluorinated phosphate, soy sprat, sunflower sprat, fish meal, limestone flour, feed yeast, vitamins and minerals, sunflower oil, antioxidant.

Supplementary Table S2. The number of rats in each experimental group.

Assay	Experimental groups					
	AL	IF	DR	BDL+ AL	BDL+ IF	BDL+ DR
Biochemical analysis of the blood	6	5	4	5	4	4
Morphological studies	6	5	4	5	4	4
Hydroxyproline level	6	N/D	N/D	5	4	4
Western blot (p62)	3	4	4	N/D	N/D	N/D
Western blot (αSMA)	3	N/D	N/D	3	3	3
Glycogen level	4	4	4	N/D	N/D	N/D
Glucose-6-phosphatase activity	6	4	4	N/D	N/D	N/D
Glycolysis enzymes activity	6	4	4	4	4	4
PDHC, TCA cycle enzymes activity, COX	6	4	4	4	4	4
TBARS, basal level	6	4	4	4	4	4
TBARS, Fe^{2+}/ascorbate-induced level	6	4	4	4	4	4
Reduced glutathione level	6	4	4	4	4	4
Activity of glutathione metabolism enzymes	6	4	4	4	4	4
Total SOD and catalase activity	6	4	4	4	4	4

Notes:
* - the value of "n" may be less than the number of rats indicated in the table for the BDL+AL group due to the presence of outliers estimated by the Grubbs test.
N/D - not determined.

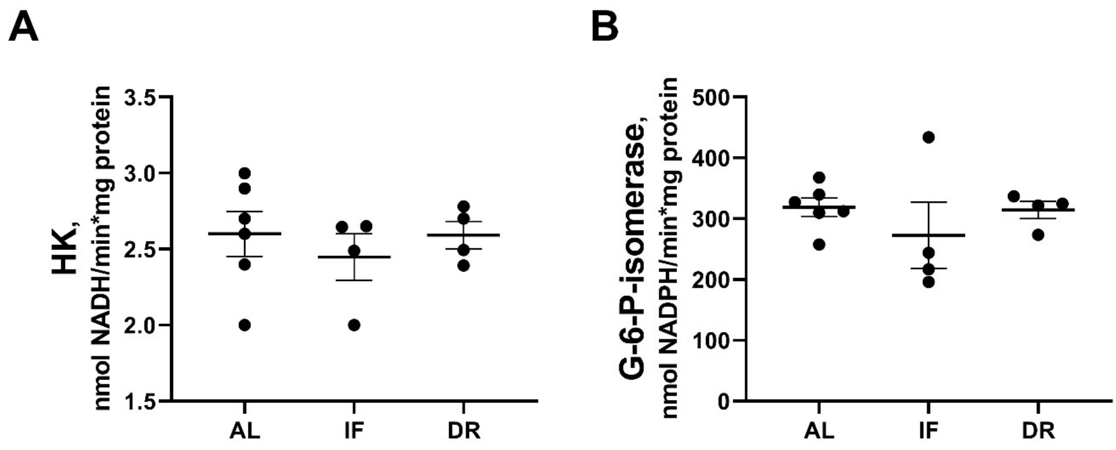

Supplementary Figure S1. Activity of initial glycolytic enzymes in the cytoplasmic fraction of the liver of rats kept on an ad libitum (AL) diet, intermittent fasting (IF) and dietary restriction. (**A**) Activity of hexokinase (HK). (**B**) Activity of glucose-6-phosphate isomerase.

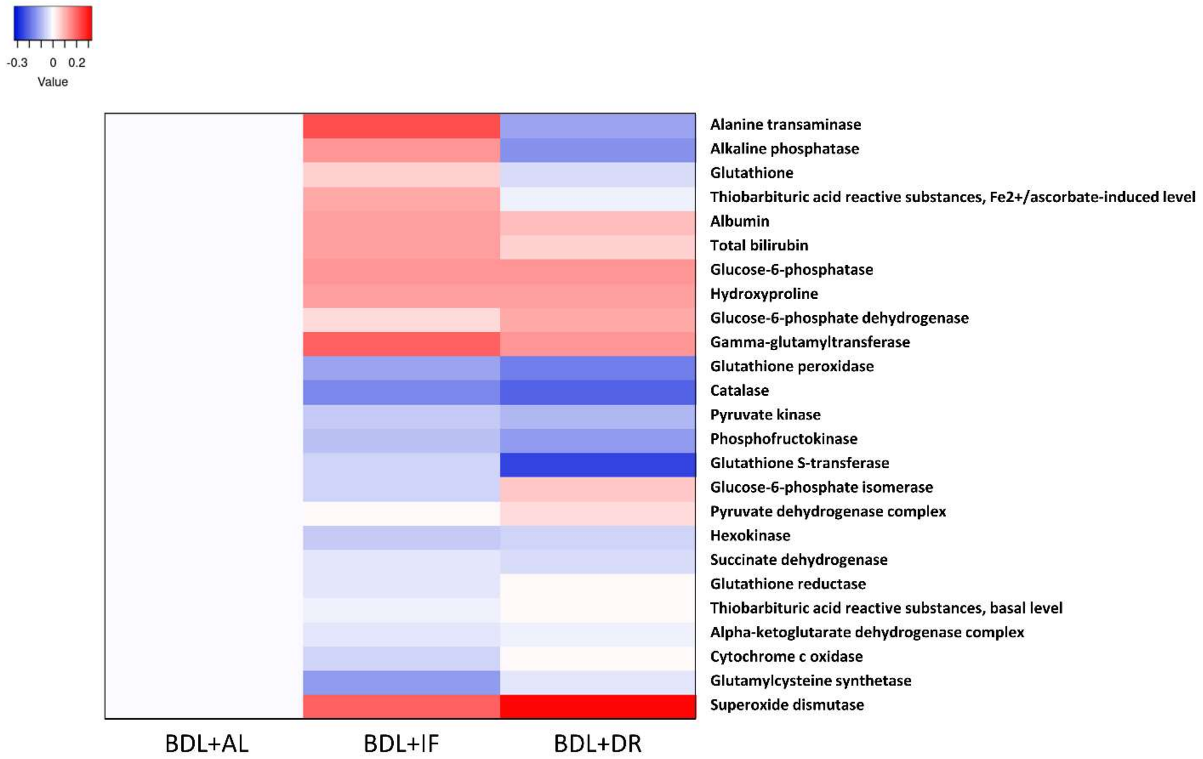

Supplementary Figure S2. Heat map of changes in biochemical parameters in rats with common bile duct ligation on an *ad libitum* diet, intermittent fasting and dietary restriction.

Supplementary Table S3. Composition of the reaction mixture for determining enzyme activity in rat liver tissue homogenates.

Enzymes	Composition of the reaction mixture	Sample dilution for assay	Diluted sample to reaction mixture ratio (v/v)	Protocol	Reference
A. Enzymes of antioxidant systems					
Catalase	50 mM potassium phosphate buffer, pH 7.4, 20 mM H_2O_2	1:800	1:10	Incubation 10 min (37 ^{0}C). Stop the reaction with a 4-fold (v/v) excess of 4%(w/v) ammonium heptamolybdate. Absorbance measurement at 405 nm. The calculations were made according to the calibration curve using a solution of hydrogen peroxide (5-20 mM).	Shangari N., O'Brien P.J., 2006
Glutathione reductase	100 mM potassium phosphate buffer, pH 7.4, 1 mM EDTA, 0.5 mM NADPH, 1 mM GSSG.	N/D	1:10	Absorbance measurement at 340 nm on a plate spectrophotometer at 37 ^{0}C for 3-5 minutes. Calculations were performed using the NADPH absorption coefficient of 6.22 mM-1*cm-1, and for CDNB-glutathione conjugate - of 9.6 mM-1*cm-1.	Mannervik, B., 2001
Glutathione peroxidase	100 mM potassium phosphate buffer, pH 7.0, 1 mM EDTA, 1 mM GSH, 1 mM NaN_3, 0.2 mM NADPH, 0.2 mM H_2O_2, 0.2 IU/ml glutathione reductase	1:80	1:10		Flohé, L.; Günzler, W.A., 1984
Glutathione-S-transferase	100 mM potassium phosphate buffer, pH 6.5, 1 mM EDTA, 0.5 mM CDNB, 1 mM GSH.	1:80	1:10		Habig, W.H., Jakoby, W.B., 1981
Superoxide dismutase	200 mM sodium carbonate buffer, pH 10.6, 0.26 mM epinephrine hydrochloride	1:80	1:20	Absorbance measurement at 340 nm with a plate spectrophotometer at 37 ^{0}C for 3-5 minutes.	Misra, H.P.; Fridovich, I., 1972; Ivanova, M.A. et al., 2022

B. Glucose Metabolism Enzymes					
Glucose-6-phosphatase	100 mM sodium citrate buffer, pH 6.5, 80 mM glucose-6-phosphate sodium salt	1:4	1:1	Incubation 15 min (37 ^{0}C). Stop the reaction by adding 10% (w/v) TCA at a ratio of 1:1 (v/v). Centrifugation for 5 minutes at 10,000 g (+4^{0}C). Measurement of inorganic phosphate (Pi) was performed in the supernatant, and the amount (nmol) of Pi formed was calculated using the calibration curve.	Baginski E.S., Foà P.P., Zak B., 1974; Lanzetta P.A. et al., 1979.
Glucose-6-phosphate dehydrogenase	100 mM glycylglycine buffer, pH 7.4, 1 mM glucose-6-phosphate, 10 mM $MgCl_2$, 100 mM KCl, 0.7 mM $NADP^+$.	N/D	1:10	Absorbance measurement at 340 nm with a plate spectrophotometer at 37 ^{0}C for 3-5 minutes. Calculations were performed using the NAD(P)H absorption coefficient of 6.22 mM-1*cm-1.	Dror Y. et al., 1970
Hexokinase	50 mM glycylglycine buffer, pH 7.4, 1 mM D-glucose, 10 mM $MgCl_2$, 100 mM KCl, 0.5 mM $NADP^+$, 5 mM ATP, 0.2 IU/mL glucose-6-phosphate dehydrogenase.	N/D	1:10		Pilkis S.J., 1975
Glucose-6-phosphate isomerase	50 mM Tris-HCl buffer, pH 8.0, 0.5 mM EDTA, 100 mM KCl, 10 mM $MgCl_2$, 5 mM fructose-6-phosphate, 0.5 mM $NADP^+$, 0.2 IU/mL glucose-6-phosphate dehydrogenase.	1:80	1:10		Gracy R.W., 1982
Phosphofructo-kinase	50 mM Tris-HCl buffer, pH 8.0, 0.5 mM EDTA, 1 mM DTT, 5 mM $MgCl_2$, 100 mM KCl, 0.5 mM NADH, 5 mM fructose-6-phosphate, 5 mM ATP, 5 mM PEP, 1 IU/ml LDH, 1 IU/ml PK.	1:80	1:10		Kemp R.G., 1975
Pyruvate kinase	50 mM Tris-HCl buffer, pH 7.4, 100 mM KCl, 10 mM $MgCl_2$, 2 mM ADP, 0.8 mM PEP, 0.5 mM NADH, 1 IU/ ml LDH.	1:40	1:10		Cardenas J.M., 1982

<table>
<tr><td colspan="6">C. Enzymes of energy metabolism</td></tr>
<tr><td>Pyruvate (or α-ketoglutarate) dehydrogenase complex</td><td>50 mM potassium phosphate buffer, pH 7.4, 2 mM $MgCl_2$, 0.1 mM CoASH, 0.2 mM TPP, 2 mM NAD^+, 1 mM Cys, 2.5 mM sodium pyruvate (or sodium α-ketoglutarate), 0.6 mM INT, 0.065 mM PMS.</td><td>N/D</td><td>1:20</td><td rowspan="2">Absorbance measurement at 500 nm with a plate spectrophotometer at 37 ^{0}C for 3-5 minutes. Calculations are carried out using the formazan absorption coefficient of 12.4 mM-1*cm-1.</td><td>Hinman L.M., Blass LI.P., 1981</td></tr>
<tr><td>Succinate dehydrogenase</td><td>100 mM Tris-HCl buffer, pH 7.4, 0.5 mM EDTA, 2 mM NaN_3, 10 mM sodium succinate, 0.6 mM INT, 0.065 mM PMS.</td><td>N/D</td><td>1:20</td><td>Munujos P. et al., 1993</td></tr>
</table>

Notes:
Reagents were obtained as follows: Tris base, coenzyme A (CoASH) lithium salt, phenazine methosulfate (PMS), β-nicotinamide adenine dinucleotide oxidized (NAD) and reduced (NADH), glutathione reductase and glucose-6-phosphate dehydrogenase from baker's yeast, 30% hydrogen peroxide solution, magnesium chloride hexahydrate, sodium α-ketoglutarate, sodium pyruvate, sodium succinate, and sodium azide from Sigma-Aldrich (Merck, Darmstadt, Germany); reduced glutathione (GSH), oxidized glutathione (GSSG), β-nicotinamide adenine dinucleotide phosphate oxidized (NADP), reduced (NADPH), and trichloroacetic acid (TCA) from AppliChem GmbH (Darmstadt, Germany); L-cysteine hydrochloride (Cys), lactate dehydrogenase (LDH) from pig muscle, iodonitrotetrazolium chloride (INT), malachite green, pyruvate kinase from rabbit muscle, and phospho(enol)pyruvic acid tri(cyclohexylammonium) salt (PEP) from Reanal (Budapest, Hungary); thiamine pyrophosphate (TPP), adenosine 5′-diphosphate (ADP), adenosine 5′-triphosphate (ATP), D-glucose-6-phosphate sodium salt, and D-Fructose-6-phosphate from Santa Cruz Biotechnology (Dallas, TX, USA). All reagents were of analytical grade, HPLC grade, or the best available pharmaceutical grade. All solutions were prepared using Milli-Q-purified water.
Samples of the cytosolic fraction were used to determine the activity of antioxidant enzymes and enzymes of glucose metabolism (except glucose 6-phosphatase). The procedure for obtaining the crude cytosolic fraction from the liver is described in the section 2.5 of the main text.
The activity of glucose-6-phosphatase, as well as mitochondrial multienzyme complexes (pyruvate dehydrogenase, alpha-ketoglutarate dehydrogenase), and succinate dehydrogenase was determined in homogenates (1:10, w/v) after sedimentation of cell nuclei and debris. The homogenization procedure is described in the section 2.5 of the main text.
N/D - not diluted sample.
